# Perceptions of eating disorder diagnoses and body image issues in four male cases in Singapore

**DOI:** 10.1186/s40337-017-0159-x

**Published:** 2017-10-20

**Authors:** Evelyn Boon, Kelly Ann Zainal, Stephen W. Touyz

**Affiliations:** 1 Department of Psychiatry, Singapore General Hospital, 226 Outram Road, Singapore, 169039 Singapore; 2 School of Psychology, University of Sydney, Sydney, Australia

**Keywords:** Male, Case report, Body image, Eating disorder, Anorexia nervosa, Bulimia nervosa, Binge eating disorder

## Abstract

**Background:**

Despite the increasing number of patients presenting for treatment, little is still known about male eating disorders cases. The current study presents four male eating disorder cases presented to our specialized treatment facility in Singapore.

**Case presentation:**

Cases 1, 2 and 3 are homosexual males in their twenties and thirties who presented with anorexia nervosa and bulimia nervosa. Case 4 is a heterosexual male in his twenties diagnosed with binge eating disorder. All four cases expressed body image dissatisfaction, fat phobia and fear of weight gain. Additionally, all of them sought treatment because of comorbid psychiatric conditions or parental wishes. Premorbid obesity and homosexual orientation may be potential risk factors for males in developing eating disorders.

**Conclusions:**

These findings suggest that more exploration needs to be done for males diagnosed with eating disorders, particularly in the Asian society. A deeper understanding into factors associated with symptom presentation and treatment-seeking behaviors would greatly assist in informing the direction and focus of treatment in the region.

## Background

To date, available literature on males with eating disorders (EDs) and their body image disturbances is sparse compared to that of females [1]. A review by Raevuori et al. highlighted several documented differences between males and females with eating disorders in terms of risk factors, clinical presentation and comorbidity [2]. Although more male eating disorder cases have been documented, the number of cases is still small relative to females and their presentation and body image disturbances still largely inconsistent and unclear [3–6]. Although there have been several qualitative studies on the male clinical population [7–11], to the best of our knowledge, only two report on male eating disorder cases have been documented in Asia, [7, 12]. In Singapore, whilst the ratio of male to female cases in the clinical sample is 1:12.5 [4], estimates of male cases in the population is posited to be higher. Female stereotypes of eating disorders still plague the identification, diagnosis and treatment in males [11, 13]. There is, therefore, an urgent need to seek better understanding of eating disorders in males [11, 13].

Limitations of previous studies include the focus on quantitative clinical presentation in male patients with eating disorders [4], as well as that on non-clinical samples [14, 15]. Moreover, even though the number of reported eating disorders cases has been increasing in Asia, available data is still lacking [16, 17]. In the present study, we aim to provide a deeper understanding into male eating disorders in Singapore by describing four male cases of eating disorders from our local clinical sample. We seek to highlight the development of their eating disorders, symptom presentation and body image disturbances. Ultimately, we hope to point out potential risk factors for eating disorders in males, and motivating factors for them to seek treatment.

## Method

All male patients were identified and recruited from a specialized eating disorders clinic in a restructured government hospital in Singapore between 2011 and 2012 with four consenting to the study and being interviewed. Ethics approval has been obtained from SingHealth Institutional Review Board and University of Sydney as part of a larger study. Informed consent has also been obtained from all four participants involved in this study.

Retrospective medical records review was carried out for all participants involved in this study, in which demographics and clinical characteristics were compiled and analyzed. E.B. conducted semi-structured interviews with all four participants, using questions as shown in Table 1, as well as other probing questions to obtain more in-depth information about their eating disorders. Participants were interviewed individually in a quiet room within the clinic. All interviews were voice-recorded with participants’ consent. Following the interviews, K.A.Z. listened to the recorded interviews in detail and transcribed them verbatim for documentation. Both E.B. and K.A.Z. ensured the accuracy of the transcription by checking the transcripts against the recordings again. Refer to Table 2 for an overview of patients’ demographics and clinical characteristics.

**Table 1:** Interview schedule for semi-structured interviews

Items
a) Eating Disorders History Could you give me a brief history of how you developed eating disorders? How do you feel about having an eating disorder? b) Body Image Issues When did you recognize you had body image issues? What effects did it have on your life? How did you deal with how you feel about your body/body image? How do you feel about your weight and size? / body? Before you fell ill and while you were ill? (Prompts: Physically? Emotionally? Mentally?) How does how you feel about your size/weight affect your daily life? c) Effect of Body Image Issues on Illness and Recovery Does how you feel about your body affect your treatment and recovery? How does it affect it? (Prompts: Treatment? Recovery?)

**Table 2:** Demographics and clinical characteristics upon first presentation in our treatment facility

Patient ID	Ethnicity	Body mass index (kg/m^2^)	Homosexual orientation	History of obesity	Eating Disorder diagnosis at presentation	Presence of comorbid psychiatric diagnoses
C1	Chinese	16.5	Yes	No	Anorexia nervosa – restrictive subtype	Yes
C2	Chinese	18.8	Yes	Yes	Anorexia nervosa – restrictive subtype	Yes
C3	Chinese	19.5	Yes	Yes	Bulimia nervosa – purging subtype	Yes
C4	Chinese	36.8	No	Yes	Binge eating disorder	Yes

## Case presentations

### Case 1: Recovered male with a history of Anorexia Nervosa (AN)

C1 is a single Singaporean Chinese, 33, an accountant and homosexual male. He first presented for treatment in early 1990s following a suicide attempt, and was diagnosed with Anorexia Nervosa – Binge-Purge subtype (AN-BP) and comorbid Major Depressive Disorder (MDD), later re-diagnosed as having Bipolar Disorder. Dependent personality traits later emerged in the course of his treatment. He, an only child, was brought up by his maternal grandparents, as his mother had passed away when he was fifteen and his father had schizophrenia.

For C1, his ED seemed to have been triggered by a failed relationship, in which his first boyfriend could not accept being homosexual. Initially, he was treated predominantly for depression and relationship issues. It was only when he lost 20 kg in five months when he was 25, that he was referred to an eating disorder specialist. His weight loss started after noticing his weight of 70 kg (Body Mass Index (BMI) 22) and receiving comments from friends regarding his weight gain. He said it had “*freaked*” him out so he “*just started exercising, started to binge-purge and stuff like that*”. His relationship problems often worsened his restriction, resulting in a total of 10 hospital admissions. He was very open to psychotherapy and treatment, and had been in treatment since 2003.

Although he was never overweight throughout his childhood, he displayed fat phobia and prominent body image issues. He also had intense fear of being fat, reporting that fat people triggered and scared him immensely and that he harboured negative stereotypes of being overweight. He stated that “*[being overweight] spoils my whole image*”, “*freaks me out*”, and added that “*It’s like you can’t wear nice clothes. And you have to eat a lot and sweat a lot and kind of stinky*”. He went further to say that if he were to become overweight one day, “*[he] would just kill [himself]*”. His fear of being fat seemed to have stemmed from childhood, as his mother was taking diet products to lose weight.

C1’s ED has significantly impacted his life – causing delays to his education, missing milestones of his adult life like career and relationships. Despite being 33, C1 still seemed regressed, speaking and acting much like someone in his twenties. He reflected that his main barriers to recovery were the fear of the unknown of being well, being beyond what he deems as his ideal weight and the fear being fat. C1 is still in treatment for the maintenance of both ED and Bipolar Disorder. Despite reaching healthy weight range he still struggled with his body image issues and continues purging once or twice a week. Focus of treatment now is to maintain normal eating and relationship issues.

### Case 2: Male recovered from Bulimia Nervosa (BN)

C2 is a single 26-year-old Chinese Singaporean homosexual male. He had recently graduated from University and is awaiting entry into a post-graduate course in Clinical Psychology. He is the eldest of two children, born into a middle class dual-income family. C2 first presented for treatment in 2003 at 15 years of age for extreme weight loss.

Being overweight throughout childhood, C2 was enrolled in the Trim and Fit Club, a local school-based weight management program targeted at increasing physical activity and reducing childhood obesity rates [18], during his primary and secondary school days. He reported hating everything about the club, feeling shamed and humiliated by it. He started dieting and exercising at age 13 upon discovering that he had high blood pressure. At presentation he was at his lowest weight of 52 kg, with BMI 18.8 kg/m^2^. He reported feeling happy and guilty at the same time upon seeing his weight. Shortly after first presentation, his weight plummeted to 45 kg, starting a series of hospital admissions (he also absconded from hospital on several occasions) and an admission to a mental health institution. C2 has been receiving individual and family psychotherapy since 2006. During the course of treatment, his diagnostic picture changed from that of AN to BN.

C2 has reported that though his initial weight loss was triggered by health reasons, the maintenance factors are his looks and negative stereotypes of being fat. To him, being fat represents “*[not having] enough self-control*”, being “*sloppy, overweight, eat doughnuts all day, kind of smell funny, nerdish*” and he “*would do anything in [his] power to not get there*”. In terms of body image issues, he found himself realizing he was underweight, but “*all that crowded out when [he focuses] on a body part*”. His preferred body shape was one of muscularity, and not thinness- or weight-driven.

C2 described receiving the diagnosis and the impending treatment ambivalently as “*a bit of a relief because it’s like someone actually knows me*” and “*a loss of control*” respectively. He was initially resistant to therapy and recovery and described his main fears as being loss of control and the fear of becoming fat again.

Through years of outpatient individual psychotherapy, C2 has since recovered from BN and came to terms with his body shape and size. He found mindfulness to be crucial in helping him feel connected again and come to peace with his body. He continued with psychotherapy to deal with his social anxiety and depression, as well as the residual ED symptomatology which often surfaces as a coping strategy during transitions or periods of anxiety and stress. He was discharged from treatment in 2015, now fully recovered from ED and on his way to study Clinical Psychology.

### Case 3: Male with BN

C3 is a 28-year-old single Chinese homosexual male who is currently working as an assistant manager. He is the second of three children. His parents are in their 50s – father an investor and mother a cashier. He has done well in his studies, graduated with a degree in Business Management. He first presented for treatment in 2002 and was diagnosed with BN – Purging subtype, Bipolar II Disorder, Alcohol Dependence Syndrome and Substance Abuse He defaulted after only one session, but subsequently resumed treatment in 2009.

C3 developed disordered eating during his National Service, a compulsory conscription to the army for two years for all males. At the time of presentation to the specialist clinic, he started purging after receiving comments about his weight gain. In 2009, his eating and purging had increased in both intensity and frequency due to relationship problems with his boyfriend. He was a moderate smoker and heavy drinker, with an intake of up to 80 units per week. In addition, he abused substances like Phentamine and Alkyl Nitrites regularly and Cocaine and 3,4-Methylenedioxymethamphetamine (MDMA) occasionally to ‘get high’. At this point, he was 57 kg with a BMI of 19. His heaviest weight was 80 kg (BMI 27) and lowest at 53 kg, and his ideal weight being 55 kg (though the lower the better). He was treated primarily with medication and outpatient consultations with a psychiatrist, declining psychotherapy and hospitalization.

C3 had significant dissatisfaction with his body and appearance, feeling self-conscious about his looks and fat each time he looked in the mirror. In terms of body image, C3 admitted that his sexual orientation could have played a part in what he perceived as a perfect body, that being thin dictates his attractiveness within the gay community. To him, “*[being slim] is very important, all gays are slim… Fat gays are always with fat people. And none of the supermodels are fat, you know. So that sets the model person for you to want to be like*”, and that this is a common problem amongst his friends. Furthermore, C3 has a preoccupation with going past the 60 kg mark, stating that “*[he’d] get ‘mang zhang’ (it’s a Chinese term for frustrated, irritated)*”.

ED had negatively affected C3’s social functioning. He not only reported being more withdrawn from friends, but his urge to purge was so strong despite consequences such as missing classes and arousing suspicions from superiors at work.

C3 denied any barriers to recovery, stating he was very keen for treatment as he felt the medications helped control his mood swings and binges and hence keep his weight down. Throughout the recovery process C3 had only seen his psychiatrist, and was on medications for Bipolar Disorder and BN. His binge-purge symptoms still persist, but he was nonchalant about it and seemed satisfied in remaining status quo. He has since followed his psychiatrist to another treatment centre.

### Case 4: Male recovered from Binge Eating Disorder (BED)

C4 is a single 20-year-old Chinese heterosexual male who had been diagnosed with Obsessive Compulsive Disorder (OCD) and Attention Deficit Disorder (ADD) when he was younger. C4 has an older sister and is the youngest in the family. His parents are both medical professionals. There was a strong familial history of psychiatric disorder on his paternal side, as his uncles had ADD and Obsessive Compulsive Personality Disorder, and father had MDD. C4 first presented for treatment in 2011 due to significantly increasing weight from binge eating at 110 kg and BMI 36, his heaviest weight to date.

Shortly before presenting to our treatment facility, C4 was diagnosed with OCD by a private psychiatrist; he would shower excessively and wash his beddings daily. His OCD was more focused on contamination, and some checking behavior. He was diagnosed with ADD at the age of four and was started on Ritalin at around age 7. His parents reported noticing a change in C4’s eating upon starting Ritalin; he would have no appetite during school hours but eat two or three portions of meal when he got home. C4 himself reported that his BED may have started when he was 12 years old as deteriorating poor eating habit, denying any other trigger or significant event.

To him, the disordered eating was never a problem, though the resulting increasing weight was. He dismissed the diagnosis of having BED as “*just a mere label*” and “*did not want it to be an excuse for [his weight]*”. He denied having any significant body image issues, even at his heaviest but admitted being mildly dissatisfied with his body as he “*[prefers] to have a better figure*”. C4’s life seemed to have been minimally impacted by his BED, likely because he had never felt he had an ED and had always attributed his eating to being “*just greedy*”. He also seemed to have a relatively intact and strong ego and self-esteem.

Overall, C4 seemed keen on treatment and did not seem too bothered by his hospitalization and recovery. He stopped individual psychotherapy as he was symptom-free for his BED and OCD, and had also reached a stable healthy weight range. He is still on follow up with his psychiatrist.

## Discussion and conclusion

To date there was only one study on males with eating disorders in Singapore by Tan et al., a retrospective descriptive cross-sectional investigation of the male clinical population [4]. It had contributed to the knowledge of males with eating disorders in Singapore by giving them a clearer representation in the local eating disorders literature. This study hopes to add to the depth and breadth of knowledge as it offers insights into these four males and their relationship with their conditions.

All four patients interviewed expressed fat phobia and significant fear of weight gain and body image dissatisfaction. For three of the patients (excluding C4), the fear of weight gain was a strong deterrent to recovery. The numbers on the scale were a point of concern and focus for all four of them. The aforementioned factors were similarly found in Tong et al.’s clinical sample of males in China, as well as Tan et al.’s case series from Singapore [4, 6]. Tan et al. found that 68% of male patients with eating disorders reported body dissatisfaction [4].

Homosexual orientation and premorbid overweight are documented risk factors for eating disorders in males [2]. Tan et al. also found that 15.3% of the male clinical sample in Singapore were homosexual/bisexual, and 59.7% reported premorbid obesity [4]. All except C1 was overweight or obese prior to the onset of their ED; even for C1, who was never really overweight, was triggered into ED because of weight gain and comments from peers that he was becoming fat. This finding echoed the known risk factor of premorbid obesity in males. The other known risk factor was homosexual orientation. In our clinical sample, three out of four interviewed were of homosexual orientation. Some even specifically cited that their sexual orientation did impose some body shape and size concerns onto them. A few theories have been proposed to explain the higher prevalence for eating disorders in homosexual males, however the consensus seems to be that there is a heightened focus on physical appearance in the gay community, giving rise to peer pressure for homosexual individuals to conform [19–21]. The drive for thinness or muscularity, however, is posited to parallel measures of femininity and masculinity as opposed to sexual orientation in the community and clinical samples [20, 22]. An interesting point to note is that while C1’s ideal figure is slim and C2’s is muscular, C3 focuses strongly on the number on the scale, citing that he is preoccupied with not exceeding the 60 kg mark. Males with eating disorder are known to possess higher reluctance to seek treatment than females and therefore go undiagnosed, with perceived stigma to seek treatment being an important contributing factor. For eating disorders, this may be exacerbated by the perception that eating disorders are “female” problems, or is something they should cope with on their own [23, 24]. In our sample, the impetus to seek treatment did not seem to stem from having an ED, but rather a comorbid condition for C1 and C3, or having parents who brought C2 and C4 for treatment. It was not known if they felt stigmatized to seek help for their ED, did not realize they had ED, or any other reasons. For C4, it was clear he did not and still do not see his BED as a disorder, but instead as a mere label. However, more needs to be researched upon to explore the detailed reasons for such treatment delays.

The main strength of this study is that it is, to our knowledge, the first to report clinical cases of men with eating disorders in the Singapore population. However, we do recognize limitations such as the small sample size that prevented this study from becoming a full qualitative investigation into how males perceive and experience ED. This would also mean that this study’s findings may not be representative nor could it be generalized. To gain more insight and deeper understanding of the male eating disorder, we would need to replicate this case report with a qualitative study with a larger and more representative sample. Due to the dearth of literature in males with eating disorders in Singapore, it is crucial to conduct qualitative and quantitative studies to allow for more in-depth comparisons with females in the clinical population, as well as males in ‘Western’ countries. This would greatly assist in informing the direction and focus of treatment of males with eating disorders in the region.
